# Health of the Nation Outcome Scales for Infants field trial: concurrent validity

**DOI:** 10.1192/bjo.2021.951

**Published:** 2021-07-12

**Authors:** Peter Brann, Gordana Culjak, Nick Kowalenko, Rosemary Dickson, Tim Coombs, Philip Burgess, Anne Sved Williams, Elisabeth Hoehn, Margaret Hoyland

**Affiliations:** Eastern Health Child Youth Mental Health Service and Adjunct Lecturer Monash University, Australia; Australian Mental Health Outcomes and Classification Network (AMHOCN), Sydney, Australia; and Health Education and Training Institute (HETI), Sydney Medical School, University of Sydney, Australia; Department of Psychological Medicine, Saunders Unit – Mental Health, Children's Hospital Randwick, Sydney Children's Hospital Network, Australia; and Sydney Medical School, University of Sydney, Australia; Child and Adolescent Mental Health Information Development Expert Advisory Panel (CAMHIDEAP) Secretariat, Australian Mental Health Outcomes and Classification Network (AMHOCN), Health Education and Training Institute (HETI), Australia; Australian Mental Health Outcomes and Classification Network (AMHOCN), Sydney, Australia; and Health Education and Training Institute (HETI), Australia; Australian Mental Health Outcomes and Classification Network (AMHOCN) Analysis and Reporting, Sydney, Australia; and School of Public Health, Faculty of Medicine, The University of Queensland, Australia; University of Adelaide and Consultant Psychiatrist, Women's and Children's Health Network, Australia; Queensland Centre for Perinatal and Infant Mental Health Child and Youth Mental Health Service (CYMHS), Children's Health Queensland Hospital and Health Service, Australia; Child and Youth Mental Health Service (CYMHS), Children's Health Queensland Hospital and Health Service, Australia

**Keywords:** HoNOSI, mental health, outcome measure, infants, validity

## Abstract

**Background:**

A review of Australian mental health services identified a gap in routine outcome measures addressing social, emotional and behavioural domains for pre-schoolers and infants. A Child and Adolescent Mental Health Information Development Expert Advisory Panel working group developed the Health of the Nation Outcome Scales for Infants (HoNOSI), a clinician-reported routine outcome measure for infants 0–47 months. Prior face validity testing showed that the HoNOSI was considered useful in measuring mental health outcomes.

**Aims:**

To examine the concurrent validity of the HoNOSI.

**Method:**

Mental health clinicians providing assessment and treatment to infants in routine clinical practice participated in the study. The mental health status of 108 infants were rated by a minimum of 26 clinicians with the HoNOSI, the Parent-Infant Relationship Global Assessment Scale (PIR-GAS) and measures of symptom severity and distress.

**Results:**

The HoNOSI was statistically significantly correlated with the PIR–;GAS, *r_s_* = −0.73; Clinical Worry, *r_s_* = 0.77; and Severity Judgement ratings, *r_s_* = 0.85; *P <* 0.001. A good level of internal consistency was found. Using the COsensus-based Standards for the selection of health Measurement INstruments (COSMIN) criteria for judging instrument acceptability, the HoNOSI meets the standard for both concurrent validity and internal consistency.

**Conclusions:**

There has been a clear need for a routine outcome measure for use with infants. This study provides positive evidence of aspects of validity. These findings, along with those from the prior face validity study, support a controlled release of the HoNOSI accompanied by further research and development.

## Background

In 1990 Jenkins identified an urgent need for a system of indicators to enable clinicians to monitor and evaluate mental healthcare.^1^ One reason identified for not routinely using standard outcome measures was the lack of appropriate instruments.^2^

In 1998, Wing and colleagues^3^ developed the Health of the Nation Outcome Scales (HoNOS), an instrument covering symptoms, functioning, relationships and environmental issues^4,5^ that could be used routinely in the National Health Service (UK) to measure progress towards the target set by the Department of Health in the UK ‘to improve significantly the health and social functioning of mentally ill people’.^6^ Since then, the HoNOS and its adaptations for children and adolescents (HoNOSCA) and for those over 65 years of age (HoNOS65+) have been officially adopted in England, Australia, New Zealand^7^ and in other European countries.^8–10^

## HoNOSCA

Gowers et al^11^ developed the HoNOSCA, for children and adolescents, as a set of scales to be used in child and adolescent mental health services.^12^ The HoNOSCA has been widely used.^4,13–19^ It was designed to be brief, have a similar structure to the HoNOS and provide a broad, quantitative measure of severity, with sound psychometric properties, to measure a range of behavioural, symptomatic, social and impairment domains in children and adolescents.^11,20^ HoNOSCA is most appropriately applied to those over 4 years old.^21^

## Developing the HoNOSI

In Australia, the National Outcomes and Casemix Collection (NOCC) was introduced in the early 2000s ‘to provide a suite of measures that support clinical practice and comparisons across services and different consumer populations’.^19^ The Strategic Directions 2014–2024 report^22^ on the NOCC implementation and its future direction identified a gap in outcome measures for infants and pre-schoolers. The Australian Child and Adolescent Mental Health Information Development Expert Advisory Panel (CAMHIDEAP) provides advice on routine outcome measures and on information initiatives to the states, territories and the Commonwealth Government.^23^ CAMHIDEAP developed the Health of the Nation Outcome Scales for Infants (HoNOSI)^24^ as a routine outcome measure for clinicians working with the emotional and social well-being of children in the 0- to 47-month age group.

The HoNOSI arose out of an international collaboration around the reliability of the HoNOSCA.^13^ CAMHIDEAP decided the HoNOSI would parallel the structure of the HoNOSCA. A similar approach to ratings, number of scales, time frames and sources of information was considered to facilitate acceptance by clinicians who may work with both instruments. It could reduce training time. A key strategic consideration was that the adoption of a new outcome measure (especially across a nation) involves substantial financial costs associated with database development and maintenance. A similar structure would only require the addition of a ‘version’ flag, a relatively inexpensive approach, in order for HoNOSI ratings to be recorded and extracted from the existing HoNOSCA data space.

The content of the 15 scales was initially developed by Dr Sally Merry of New Zealand, with in principle support from key figures of the HoNOSCA reliability collaboration from the UK, Denmark, Norway and Australia. Dr Merry, with support from infant and child mental health colleagues, either paralleled HoNOSCA scales where appropriate, or replaced them with more developmentally appropriate areas of concern. Continuity of outcomes could be assisted by maintaining both structural similarity and maximising content overlap where appropriate.

Face validity testing^25^ showed that the HoNOSI fulfilled a much-needed gap in infant mental health outcome measurement for the 0- to 47-month age group as no suitable instrument previously existed. Following face validity testing, the CAMHIDEAP working group identified the need for field testing to test selected psychometric properties of the HoNOSI.

## Aim

The HoNOSI field trial was designed to examine concurrent validity – how well HoNOSI ratings correlate with other measures of similar constructs.

## Method

The CAMHIDEAP nominated key clinicians across a range of Australian states who were engaged in providing mental health services to infants and pre-schoolers. These key clinicians approached their own and allied services that provided infant mental health services. Many of these clinicians had previously been involved in the face validity study.^25^ Services from states previously participating in the face validity study were invited to participate from Queensland, New South Wales, Victoria and South Australia.

Concurrent validity was assessed with patients in routine clinical care by comparing clinician's ratings on the HoNOSI against the Parent-Infant Relationship Global Assessment Scale (PIR-GAS), Clinical Worry Rating scale and Severity Judgement Rating scale (see Psychometric properties tested section below for further details). Each infant was rated by one clinician on each of these four measures. Data was collected from five participating services across four states. Participants were given an overview of the study, including rationale, background and aims and were provided with an information sheet that they were asked to read, before signing a consent form in order to be able to participate. Site coordinators emphasised that the clinician's information would remain confidential and be analysed in aggregate, anonymous form only. Copies of the study protocol were included with the study material for co-ordinators’ and participants’ reference.

Instructions for using the HoNOSI, PIR-GAS, Clinical Worry and Severity Judgement Rating scales were included with the instruments. Additional background material and principles for rating as well as the glossary for each scale were incorporated into the HoNOSI. Participants were encouraged to ask any questions of the site co-ordinator or the project co-ordinator.

Signed consent forms and completed ratings were returned via courier to the Health Education and Training Institute for data input and analysis. In the rare event that the clinician returned a completed rating scale(s) without having completed a consent form, consent was implied via the participant's return of the completed outcome rating. No information was sought from children, infants or parents. Ethics and site-specific approval were obtained from the respective Ethics and Research and Governance Offices within each participating state – New South Wales, Queensland, Victoria and South Australia.

### Psychometric properties tested

The COnsensus based Standards for the selection of health Measurement INstruments (COSMIN)^26^ initiative was developed to provide guidance on the selection of high-quality patient-reported outcome measures to clinical and research applications.^27^ This includes providing a methodology for assessing the content validity of patient-reported outcome measures.^26,28^ It comprises a taxonomy and definitions of measurement properties,^29^ checklists for assessing the methodological quality of measurement properties^30^ and criteria for good measurement properties, against which to evaluate study results.^27^

The minimum ‘acceptable’ COSMIN standard for internal consistency, or the degree of interrelatedness among items, is 0.70.^27^ The COSMIN standard for assessing concurrent validity, or the correlation of the measure of interest with a ‘gold standard’ is 0.70.^29^ The ‘gold standard’ is another measure, or set of measures, that assesses a similar construct.

As no single gold standard measure of clinician-rated mental health symptoms and functioning existed at that time,^31^ the working group determined that the best comparison available was to test HoNOSI^24^ against the Parent-Infant Relationship Global Assessment Scale,^32^ widely used in Germany,^33,34^ Denmark,^35^ the USA^32,36^ and Australia.^37^ As HoNOSI also covers symptom severity and perceived distress as well as functioning, two simple scales, developed by the project working group (Clinical Worry and Severity Judgement)^31^ were also rated.

### Measures

The HoNOSI^24^ contains 15 single-item scales that address a range of symptoms and functioning that can occur in the infant-to-pre-school age range (see Appendix). Each scale is accompanied by a glossary outlining the range of issues covered and is rated on a 0–4-point scale ranging from ‘*No problem*’ to ‘*Severe problem*’. The guidelines allow clinicians to include all sources of information when making a rating and do not simply presume that any difficulty is located exclusively within the infant. In parallel with the HoNOSCA, the first 13 scales cover clinical areas and are summed to form a total score. Missing data is treated as zero in calculating totals. Scales 14 and 15 focus on information about the situation^38^ and do not contribute to the total score.

Clinicians’ ratings on the HoNOS family of measures can be categorised as ‘clinically significant’ if a problem area is rated as *mild*, *moderate* or *severe to very severe* problem (i.e. a rating of 2, 3 or 4) or ‘clinically not significant’ for ratings of 0 or 1.^39^ Full details on study procedures and HoNOSI scoring instructions are available in the HoNOSI field trial report.^31^

The PIR-GAS^32^ is a measure of the quality of the parent–infant relationship.^32^ Clinicians assess the intensity, frequency and duration of difficulties on a 100-point rating scale, usually reported in deciles, that ranges from 1–10 *Documented Maltreatment* to 91–100 *Well Adapted*.

The Clinical Worry Rating,^31^ a seven-point rating scale, developed by the HoNOSI project working group, asks the clinician to rate: ‘Overall, how concerned are you about this infant?’. The Severity Judgement Rating,^31^ also a seven-point rating scale developed by the working group, asks the clinician to rate: ‘In your clinical judgement, how severe do you consider the infant's overall social and emotional problems?’. Both the Clinical Worry Rating and the Severity Judgement Rating scales were designed to be unidirectional, from 0 (*Not worried/No problem*) to 6 (*Extreme/Severe*).

Based on the directionality of the measures, the previously discussed COSMIN standards indicate that adequate concurrent validity would be achieved if the HoNOSI had a statistically significant correlation of at most negative 0.70 with PIR-GAS and at least 0.70 with the Clinical Worry and the Severity Judgement Rating scales.

### Data collection and analysis

Data was collected from five participating services within four states across Australia. The analysis dataset consisted of 108 completed clinical cases. A HoNOSI ‘item severity structure’ index was derived using the method described by Gowers et al (1999)^11^ with respect to the HoNOSCA. Statistical analyses were performed using SPSS Version 24^40^ and Stata Version 14.2.^41^

## Results

Using a combination of jurisdiction, profession and years’ experience, it is estimated that 26 clinicians participated. The number of infants rated varied from 1 to 14 with a mode of 3.5. The number of infants rated across the five sites ranged from 6 to 55 with two services being responsible for 78% of the infants rated. All statistical analyses used a type error rate of α < 0.05 and their associated probability are reported.

### Profession characteristics

Over half of the clinicians were either psychologists or social workers and these two professions completed approximately two-thirds of all ratings. Table 1 shows the estimated number of clinicians and the number of ratings completed by profession type.

**Table 1:** Infants rated by clinician's profession type

Profession	Clinicians		Infants	
	*n*	%	*n*	%
Psychologist	8	30.8	41	38.0
Social worker	6	23.1	30	27.8
Psychiatrist	4	15.4	10	9.3
Nurse	3	11.5	15	13.9
Occupational therapist	1	3.8	7	6.5
Speech pathologist	1	3.8	1	0.9
Unknown	3	11.5	4	3.7
Total	26	100.0	108	100.0

### Clinical experience

Over 61% of clinicians rating the cases had over 5 years’ experience and of those, 23% had clinical experience of over 10 years; these clinicians rated more than 75% of the cases. Table 2 shows the estimated number of clinicians and the number of ratings completed by the clinicians’ years of experience.

**Table 2:** Infants rated by clinicians’ years of experience

Clinical experience	Clinicians		Infants	
	*n*	%	*n*	%
<2 years	5	19.2	15	13.9
2–5 years	4	15.4	9	8.3
5−10 years	10	38.5	47	43.5
10+ years	6	23.1	35	32.4
Unknown	1	3.8	2	1.9
Total	26	100.0	108	100.0

### Infant characteristics

Basic demographic data were collected regarding the age and gender of the infant. There were slightly more male (52.8%) than female infants (47.2%). The age distributions differed; male infants were somewhat older than female infants, with median ages of 16 and 10 months, respectively. Table 3 shows the age distribution of the infants.

**Table 3:** Age distribution of infants (age in months)

Gender	*n*	Mean	s.d.	Minimum	Maximum	Percentile				
						10th	25th	50th	75th	90th
Male	57	18.3	14.0	1	47	3	5	16	30	38
Female	51	16.3	13.9	1	47	2	5	10	25	41
Total	108	17.3	14.0	1	47	2	5	15	26	41

On PIR-GAS, some infants were classified as *Adapted Relationship* but most had *Features of a Disordered Relationship* (PIR–GAS rating 41–80; 49.1%) or a *Disordered Relationship* (PIR–GAS rating 1–40; 42.3%).^32^ Table 4 shows the distribution of PIR-GAS ratings.

**Table 4:** Distribution of Parent-Infant Relationship Global Assessment Scale (PIR-GAS) ratings (*n* = 104)

PIR-GAS Ratings for clinical cases	%
1–10 Documented Maltreatment	0.0
11–20 Grossly Impaired	1.0
21–30 Severely Disordered	11.5
31–40 Disordered	29.8
41–50 Disturbed	15.4
51–60 Distressed	16.3
61–70 Significantly Perturbed	8.7
71–80 Perturbed	8.7
81–90 Adapted	5.8
91–100 Well Adapted	2.9
Total^a^	100.0

a. There were four non-responses to the PIR-GAS.

The majority of infants were rated as either a ‘3’ or a ‘4’ in terms of Clinical Worry (51.0%) and Severity Judgement (43.6%) on their respective seven-point rating scales, where zero denotes *Not Concerned* and *No Problem* and a score of six denotes *Extremely Concerned* and *Extremely Severe Problem* on the Clinical Worry and Severity Judgement Rating scales, respectively (Table 5).

**Table 5:** Distribution of Clinical Worry and Severity Judgement ratings (*n* = 108)

Ratings	%	Ratings	%
Clinical Worry		Severity Judgement	
Not concerned – 0	10.2	No Problem – 0	15.7
1	9.3	1	11.1
2	12.0	2	16.7
3	26.9	3	23.2
4	24.1	4	20.4
5	12.0	5	8.3
Extremely concerned – 6	5.6	Extremely severe problem –6	4.6
Total	100.0	Total	100.0

### Distribution of HoNOSI ratings

**Fig. 1:**
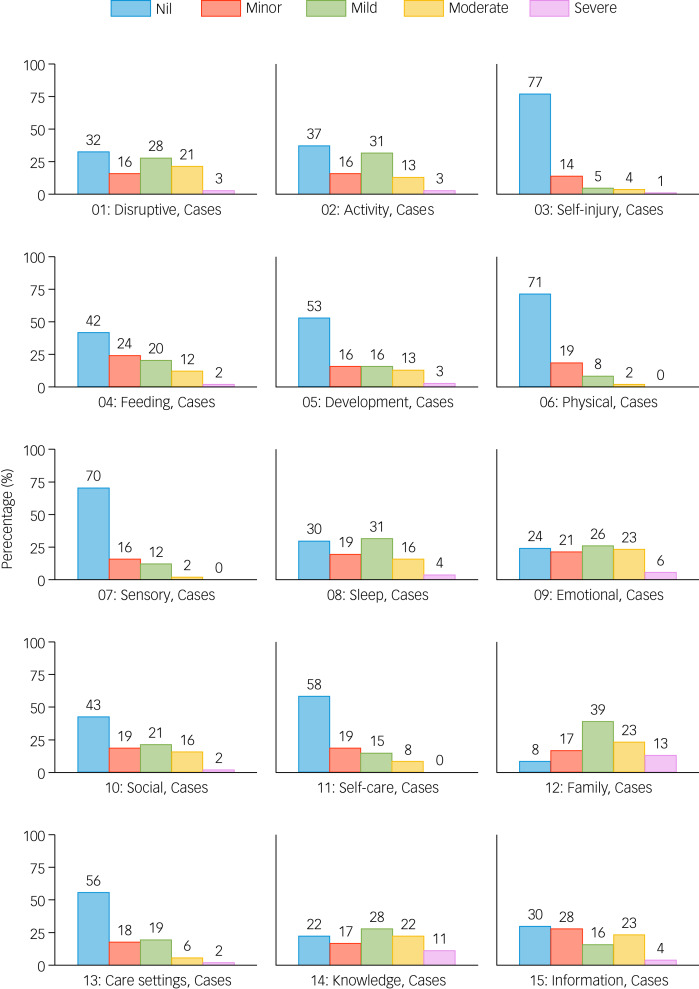
Ratings for the 15 Health of the Nation Outcome Scales for Infants (HoNOSI).

The frequency distribution of severity ratings for each of the 15 HoNOSI scales is presented in Fig. 1.

There were no missing HoNOSI ratings and no infants received a rating of *Not known/Not applicable*. Of the 108 cases, all five rating points were used for 12 of the 15 HoNOSI scales; the most severe rating of four was not used for Scale 6 *Problems with physical illness or disability*, Scale 7 *Problems associated with regulation and integration of sensory processing* and Scale 11 *Problems with age appropriate self-care and environmental exploration*. For six scales, over half of all ratings were rated zero indicating ‘*No problems/Issues*’ (Scale 3 *Non-accidental self-injury or lack of self-protective behaviours*, Scale 5 *Problems with developmental delays*, Scale 6 *Problems with physical illness or disability*, Scale 7 *Problems associated with regulation and integration of sensory processing*, Scale 11 *Problems with age appropriate self-care and environmental exploration* and Scale 13 *Problems with attending care, education and socialisation settings*).

Using the HoNOS family of measures classification where a rating of 2, 3 or 4 is classified as clinically significant, with respect to the 108 cases, 75% were rated as having clinically significant problems with Scale 12 *Problems with family life and relationships*, 55% with Scale 9 *Problems with emotional and related symptoms or over-controlled emotional regulation*, 52% with Scale 1 *Problems with disruptive behaviour/irritability/under controlled emotional regulation* and 51% with Scale 8 *Problems associated with sleep*. The scales least frequently rated as clinically significant were Scale 3 *Non-accidental self-injury or lack of self-protective behaviours* (10%), Scale 6 *Problems with physical illness or disability* (10%) and Scale 7 *Problems associated with regulation and integration of sensory processing* (14%).

Clinically significant problems were also found for 61% on Scale 14 *Problems with knowledge or understanding about the nature of the infant's difficulties* and for 43% on Scale 15 *Problems with lack of information, understanding about services, or managing the infant's difficulties*.

**Fig. 2:**
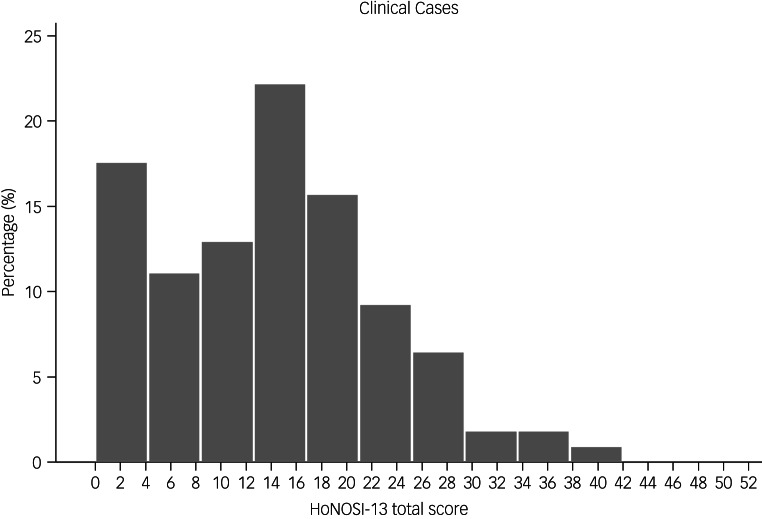
Distribution of Health of the Nation Outcome Scales for Infants (HoNOSI) total scores.

Figure 2 presents the distribution of HoNOSI total scores (sum of the ratings of the first 13 scales). HoNOSI total scores ranged from 0 through 42, with a mean and median of 14.0 and an interquartile-range of 12 points (i.e. the middle 50% of total scores were within the range 7 through 19) (Table 6). Analysis of the distribution of the total scores did not reveal any significant deviation from normality.

**Table 6:** Distribution of Health of the Nation Outcome Scales for Infants (HoNOSI) total score in clinical cases (*n* = 108)

	*n*	mean	*s.d.*	minimum	Maximum	Percentile				
						10th	25th	50th	75th	90th
Cases	108	14.0	8.8	0	42	3	7	14	19	27

The level of internal consistency of the 13 scales comprising the total score, as measured by Cronbach's ɑ is 0.87.

Collins et al (2016) address floor and ceiling effects for measures reporting total scores as ‘the percentage of respondents with the lowest possible score (floor effects) and the highest possible score (ceiling effects)’.^42^ Floor and ceiling effects are not considered statistically significant if less than 15% of participants score the lowest or the highest possible score. There was no evidence of these effects in the HoNOSI total scores (Fig. 2). Only six cases (5.6%) had a HoNOSI total score of zero and only one case had a HoNOSI total score of 42 (0.9%).

In terms of item severity structure, it is important to note that more than 80% of cases had at least one HoNOSI scale problem area rated as clinically significant. Table 7 shows details on the item severity structure index by the mean HoNOSI total score.

**Table 7:** Health of the Nation Outcome Scales for Infants (HoNOSI) item severity structure by HoNOSI total score (*n* = 108)

HoNOSI Severity Index	*n*	%	Cumulative %	HoNOSI (mean)
Two or more ratings of 4	9	8.3	8.3	27.0
Only one rating of 4	12	11.1	19.4	17.7
No 4 ratings, any ratings of 3	42	38.9	58.3	17.4
No 3 or 4 ratings, any ratings of 2	28	25.9	84.2	10.0
No 2–4 ratings, any ratings of 1	11	10.2	94.4	4.1
0 ratings only	6	5.6	100	0
Total	108	100	100	14.0

Spearman's rank order correlation was used to test the concurrent validity of the HoNOSI total score with the PIR-GAS, Clinical Worry and Severity Judgement Rating scales and the results are presented in Table 8. A comprehensive intercorrelation analysis of the 15 individual HoNOSI scales is available in Supplementary Table 1 available at https://doi.org/10.1192/bjo.2021.951. It shows that that all 15 HoNOSI scale correlations with the PIR-GAS, Clinical Worry and Severity Judgement Ratings are statistically significant (*P <* 0.001) with the one exception of Scale 6 correlated against the Clinical Worry Rating, which is also statistically significant at a lower threshold (*P <* 0.05). The HoNOSI total score correlations summary table (Table 8) shows the three validity measures correlated against the HoNOSI total score. It is also important to note that the three concurrent validity measures are highly statistically intercorrelated (*P <* 0.001): PIR-GAS with Clinical Worry, *r_s_* = −0.81; PIR–GAS with Severity Judgement, *r_s_* = −0.76; and Clinical Worry with Severity Judgement, *r_s_* = 0.81.

**Table 8:** Correlation of Health of the Nation Outcome Scales for Infants (HoNOSI) total score with Parent-Infant Relationship Global Assessment Scale (PIR-GAS), Clinical Worry and Severity Judgement rating scales

	Spearman's rho correlation coefficient^a^			
	PIR-GAS (*n* = 104)	Clinical Worry (*n* = 108)	Severity Judgement (*n* = 108)	HoNOSI total score (*n* = 108)
PIR-GAS	1.00			
Clinical Worry	−0.81	1.00		
Severity Judgement	−0.76	0.81	1.00	
HoNOSI Total Score	−0.73	0.77	0.85	1.00

a. All correlations were statistically significant (*P <* 0.001).

## Discussion

### Main findings

This study was designed specifically to establish the level of evidence of concurrent validity with respect to the 15 HoNOSI scales and the HoNOSI total severity score. In order to test concurrent validity, in the absence of a gold standard, the HoNOSI was compared with other measures that measure similar constructs: the PIR-GAS, Clinical Worry and Severity Judgement Rating scales.

The level of internal consistency of the 13 scales comprising the total score, as measured by Cronbach's ɑ, is 0.87 which well exceeds the COSMIN threshold. It should also be noted that the evaluation of the concurrent validity of HoNOSI was based on three independent measures. The three concurrent validity measures are highly statistically intercorrelated, suggesting a high degree of construct congruence. Using the COSMIN criteria, there is evidence for HoNOSI having ‘adequate’ concurrent validity, as assessed by correlations with the PIR–GAS, Clinical Worry and Severity Judgement Rating scales.

More than 80% of cases had at least one HoNOSI scale problem area rated as clinically significant. This finding suggests that the overall clinical severity of these 108 cases is likely representative of very young consumers seen in specialised public sector mental health services. This was not a sample that was symptom free.

Examining ratings of individual scales, no infants received a rating of *Not known*, nor were there any missing ratings. This suggests that all 15 scales were able to be used. The most severe rating of 4 was not used for three of the 15 scales: Scale 6 *Problems with physical illness or disability*, Scale 7 *Problems associated with regulation and integration of sensory processing* and Scale 11 *Problems with age appropriate self-care and environmental exploration*. It could be in this sample of 108 infants, that there were no cases with *Severe to very severe problem* for the HoNOSI problem areas. Alternatively, it could be that the glossary for these scales means that it is unlikely that a rating of 4 would be used. Future work could further explore these particular scales in another sample.

### Future research

Future research could also explore HoNOSI validity with respect to other domains and consumer attributes including the specific nature of presenting problems and diagnostic categories. There was relatively brief written training, embedded in HoNOSI, provided in this study. Although this brief approach may be seen to mirror what clinicians receive in real-world settings post any initial implementation,^43^ the impact of additional training on the performance of HoNOSI would be worth exploring. With the adult routine outcome measure, the type of training required, and its capacity to improve psychometric, and clinician, performance is an area of longstanding debate.^44,45^

There are other psychometric properties (for example, sensitivity to change) yet to be investigated. A face validity study,^25^ an interrater reliability study^46^ and this concurrent validity field trial have now been completed. The findings have been sufficiently encouraging to support controlled implementation of the HoNOSI.

## Data Availability

The data that support the findings of this study are available from the corresponding author (G.C.) upon reasonable request.

## Supplementary material

For supplementary material accompanying this paper visit http://dx.doi.org/10.1192/bjo.2021.951. click here to view supplementary material

## Appendix

Health of the Nation Outcome Scales for Infants

**Table:**

Scale	Description
1	Problems with disruptive behaviour/irritability/under controlled emotional regulation
2	Problems with activity levels, joint and/or sustained attention
3	Non-accidental self-injury or lack of self-protective behaviours
4	Problems with feeding and eating behaviour
5	Problems with developmental delays
6	Problems with physical illness or disability
7	Problems associated with regulation and integration of sensory processing
8	Problems associated with sleep
9	Problems with emotional and related symptoms or over-controlled emotional regulation
10	Problems with social reciprocity
11	Problems with age appropriate self-care and environmental exploration
12	Problems with family life and relationships
13	Problems with attending care, education and socialisation settings
14	Problems with knowledge or understanding about the nature of the infant's difficulties
15	Problems with lack of information, understanding about services, or managing the infant's difficulties
