# Supplementary material for: Health of the Nation Outcome Scales for Infants field trial: concurrent validity
Source: BJPsych Open. 2021 Jul 12;7(4):e129. doi: 10.1192/bjo.2021.951 (PMC8280775; doi:10.1192/bjo.2021.951)
Supplement: Supplementary file 1 [file S2056472421009510sup001.zip › S2056472421009510sup001/S2056472421009510sup001.docx]

Supplementary Table 1: Spearman’s Rho Correlation Matrix – Intercorrelation of HoNOSI Scales

The correlation matrix below shows that most correlations between the individual HoNOSI scales are statistically significant. The Grey shaded areas represent exceptions to these, which have a low correlation and are not statistically significant. Areas shaded in green show a correlation of above 0.5 and which are statistically significant (*r*=-0.73, *p*<0.01). The areas shaded in yellow show a correlation of *r>*0.6, *p*<0.01, however, these are HoNOSI total scores and their resulting statistic must be interpreted with caution as they are a sum of all HoNOSI scales 1 through 13 and as such, show a higher correlation than the individual scales, as excepted.

Supplementary Table 1: *HoNOSI Scales Intercorrelation Matrix*

| \| Spearman's rho Correlation Coefficient \| Scale 1 \| Scale 2 \| Scale 3 \| Scale 4 \| Scale 5 \| Scale 6 \| Scale 7 \| Scale 8 \| Scale 9 \| Scale 10 \| Scale 11 \| Scale 12 \| Scale 13 \| Scale 14 \| Scale 15 \| HoNOSI Total \| \| --- \| --- \| --- \| --- \| --- \| --- \| --- \| --- \| --- \| --- \| --- \| --- \| --- \| --- \| --- \| --- \| --- \| \| Scale 1: Problems with disruptive behaviour/irritability/under controlled emotional regulation \| 1.000 \|  \|  \|  \|  \|  \|  \|  \|  \|  \|  \|  \|  \|  \|  \|  \| \| Scale 2: Problems with activity levels, joint and/or sustained attention \| .541^**^ \| 1.000 \|  \|  \|  \|  \|  \|  \|  \|  \|  \|  \|  \|  \|  \|  \| \| Scale 3: Non accidental self-injury or lack of self-protective behaviours \| .413^**^ \| .453^**^ \| 1.000 \|  \|  \|  \|  \|  \|  \|  \|  \|  \|  \|  \|  \|  \| \| Scale 4: Problems with feeding and eating behaviour \| .303^**^ \| .275^**^ \| .118 \| 1.000 \|  \|  \|  \|  \|  \|  \|  \|  \|  \|  \|  \|  \| \| Scale 5: Problems with developmental delays \| .440^**^ \| .483^**^ \| .213^*^ \| .272^**^ \| 1.000 \|  \|  \|  \|  \|  \|  \|  \|  \|  \|  \|  \| \| Scale 6: Problems with physical illness or disability \| .222^*^ \| .340^**^ \| .184 \| .116 \| .253^**^ \| 1.000 \|  \|  \|  \|  \|  \|  \|  \|  \|  \|  \| \| Scale 7: Problems associated with regulation and integration of sensory processing \| .391^**^ \| .319^**^ \| .304^**^ \| .314^**^ \| .319^**^ \| .363^**^ \| 1.000 \|  \|  \|  \|  \|  \|  \|  \|  \|  \| \| Scale 8:Problems associated with sleep \| .489^**^ \| .527^**^ \| .229^*^ \| .513^**^ \| .277^**^ \| .249^**^ \| .270^**^ \| 1.000 \|  \|  \|  \|  \|  \|  \|  \|  \| \| Scale 9:Problems with emotional and related symptoms or over-controlled emotional regulation \| .452^**^ \| .559^**^ \| .422^**^ \| .288^**^ \| .406^**^ \| .257^**^ \| .519^**^ \| .416^**^ \| 1.000 \|  \|  \|  \|  \|  \|  \|  \| \| Scale 10: Problems with social reciprocity \| .337^**^ \| .568^**^ \| .220^*^ \| .416^**^ \| .463^**^ \| .235^*^ \| .402^**^ \| .432^**^ \| .614^**^ \| 1.000 \|  \|  \|  \|  \|  \|  \| \| Scale 11: Problems with age appropriate self-care and environmental exploration \| .359^**^ \| .464^**^ \| .216^*^ \| .193^*^ \| .305^**^ \| .266^**^ \| .435^**^ \| .155 \| .428^**^ \| .559^**^ \| 1.000 \|  \|  \|  \|  \|  \| \| Scale 12: Problems with family life and relationships \| .343^**^ \| .381^**^ \| .304^**^ \| .250^**^ \| .349^**^ \| .119 \| .324^**^ \| .331^**^ \| .514^**^ \| .416^**^ \| .207^*^ \| 1.000 \|  \|  \|  \|  \| \| Scale 13: Problems with attending care, education and socialisation settings \| .319^**^ \| .401^**^ \| .288^**^ \| .116 \| .369^**^ \| .230^*^ \| .294^**^ \| .267^**^ \| .316^**^ \| .361^**^ \| .284^**^ \| .370^**^ \| 1.000 \|  \|  \|  \| \| Scale 14: Problems with knowledge or understanding about the nature of the infant’s difficulties \| .470^**^ \| .573^**^ \| .341^**^ \| .338^**^ \| .387^**^ \| .235^*^ \| .368^**^ \| .455^**^ \| .471^**^ \| .496^**^ \| .427^**^ \| .611^**^ \| .481^**^ \| 1.000 \|  \|  \| \| Scale 15: Problems with lack of information, understanding about services, or managing the infant’s difficulties \| .382^**^ \| .551^**^ \| .323^**^ \| .215^*^ \| .240^*^ \| .199^*^ \| .404^**^ \| .413^**^ \| .520^**^ \| .436^**^ \| .361^**^ \| .510^**^ \| .388^**^ \| .706^**^ \| 1.000 \|  \| \| HoNOSI Total Scale 1-13 \| .694^**^ \| .769^**^ \| .468^**^ \| .534^**^ \| .641^**^ \| .399^**^ \| .593^**^ \| .651^**^ \| .771^**^ \| .743^**^ \| .567^**^ \| .586^**^ \| .545^**^ \| .690^**^ \| .601^**^ \| 1.000 \| |
| --- | --- | --- | --- | --- | --- | --- | --- | --- | --- | --- | --- | --- | --- | --- | --- | --- | --- | --- | --- | --- | --- | --- | --- | --- | --- | --- | --- | --- | --- | --- | --- | --- | --- | --- | --- | --- | --- | --- | --- | --- | --- | --- | --- | --- | --- | --- | --- | --- | --- | --- | --- | --- | --- | --- | --- | --- | --- | --- | --- | --- | --- | --- | --- | --- | --- | --- | --- | --- | --- | --- | --- | --- | --- | --- | --- | --- | --- | --- | --- | --- | --- | --- | --- | --- | --- | --- | --- | --- | --- | --- | --- | --- | --- | --- | --- | --- | --- | --- | --- | --- | --- | --- | --- | --- | --- | --- | --- | --- | --- | --- | --- | --- | --- | --- | --- | --- | --- | --- | --- | --- | --- | --- | --- | --- | --- | --- | --- | --- | --- | --- | --- | --- | --- | --- | --- | --- | --- | --- | --- | --- | --- | --- | --- | --- | --- | --- | --- | --- | --- | --- | --- | --- | --- | --- | --- | --- | --- | --- | --- | --- | --- | --- | --- | --- | --- | --- | --- | --- | --- | --- | --- | --- | --- | --- | --- | --- | --- | --- | --- | --- | --- | --- | --- | --- | --- | --- | --- | --- | --- | --- | --- | --- | --- | --- | --- | --- | --- | --- | --- | --- | --- | --- | --- | --- | --- | --- | --- | --- | --- | --- | --- | --- | --- | --- | --- | --- | --- | --- | --- | --- | --- | --- | --- | --- | --- | --- | --- | --- | --- | --- | --- | --- | --- | --- | --- | --- | --- | --- | --- | --- | --- | --- | --- | --- | --- | --- | --- | --- | --- | --- | --- | --- | --- | --- | --- | --- | --- | --- | --- | --- | --- | --- | --- | --- | --- | --- | --- | --- | --- | --- | --- | --- | --- | --- | --- | --- | --- | --- | --- | --- | --- | --- | --- | --- | --- | --- | --- | --- | --- |

**. Correlation is significant at the 0.01 level (2-tailed)

*. Correlation is significant at the 0.05 level (2-tailed)
